# Urodynamic characteristics of detrusor underactivity in women with voiding dysfunction

**DOI:** 10.1371/journal.pone.0198764

**Published:** 2018-06-20

**Authors:** Tsai-Hwa Yang, Fei-Chi Chuang, Hann-Chorng Kuo

**Affiliations:** 1 Department of Obstetrics and Gynecology, Chang Gang Memorial Hospital, Kaohsiung, Taiwan; 2 Department of Urology, Buddhist Tzu Chi General Hospital and Tzu Chi University, Hualien, Taiwan; University of Oklahoma Health Sciences Center, UNITED STATES

## Abstract

**Introduction and hypothesis:**

Voiding dysfunction has gained interest due to its high prevalence in the elderly. This study characterized bladder dysfunction in women with voiding dysfunction using video urodynamic studies (VUDS) focused on detrusor underactivity (DU).

**Methods:**

We studied 1914 women in which first-line medical treatment failed. Age, comorbidities, and urodynamic parameters were analyzed to determine the association between bladder sensation and contractility.

**Results:**

VUDS were normal in 2.9% (n = 56) of patients and showed DU in 23.1% (n = 443), detrusor hyperactivity and impaired contractility (DHIC) in 12.0% (n = 231), hypersensitive bladder in 17.0% (n = 325), detrusor overactivity (DO) in 2.6% (n = 49) and bladder outlet obstruction in 42.3% (n = 810). The mean age of patients in the DU and DHIC groups was significantly older than in women with normal VUDS and those with hypersensitive bladders (p<0.01). Decreased bladder sensation and larger cystometric bladder capacity were noted in the DU group compared to the DHIC, HSB, and DO groups. Bladder sensation was negatively associated with the bladder contractility. Bladder contractility index and voiding efficiency were lower in the DU and DHIC groups compared to the normal group.

**Conclusions:**

The bladder conditions of women with voiding dysfunction included DU, DHIC, HSB and DO. Bladder contractility index and voiding efficiency were significantly lowest in DU and DHIC groups and lower in HSB and DO groups than normal tracing group. Reduced bladder sensation was noted in DU and negatively associated with detrusor contractility.

## Introduction

Voiding dysfunction is defined as “abnormally slow and/or incomplete micturition, diagnosed by symptoms and urodynamic investigations,” by the International Continence Society (ICS) standardization and terminology committees[1]. Voiding dysfunction has been extensively studied in men, probably due to the significant prevalence of prostate hypertrophy in aged men causing bladder outlet obstruction (BOO). On the contrary, the prevalence of voiding dysfunction in women varies widely from 6.8% to 61.7% in selected populations, depending on the diagnostic criteria, and seems to increase with age[2]. One recent study reported voiding dysfunction in 7.2% of women who visited a urology office and in up to 12.8% of women with lower urinary tract symptoms (LUTS)[3].

Voiding dysfunctions in women are common, especially in the elderly and institutionalized populations. However, patients may be unaware of the symptoms and not visiting the doctor until serious complications develop, such as retention of urine, recurrent urinary tract infection, or even damage to the upper urinary tract. Due to the discordance between subjective complaints and objective findings, diagnosis of voiding dysfunction usually requires invasive urodynamic study. Voiding dysfunction can result from bladder outlet dysfunctions including anatomical and functional obstruction and the innate bladder dysfunctions. The combination of pressure flow studies and real-time urine flow and imaging studies of video urodynamic studies (VUDS) provide a comprehensive evaluation of lower urinary tract dysfunction[4].

Interest in detrusor underactivity (DU) among urologists has recently surged because it is a common condition that increases with age and is often overlooked in clinical practice. The ICS defines DU as “a contraction of reduced strength and/or duration, resulting in prolonged bladder emptying and/or a failure to achieve complete bladder emptying within a normal time span[5].” Thus, DU requires a urodynamic evaluation for diagnosis. Previous studies and reviews have discussed voiding dysfunction in women, with a focus on its relation with dysfunctional voiding characteristics[6]. The objective of the study is to determine the types of underlying bladder dysfunction in women with clinically voiding dysfunction using video urodynamic and to determine the video urodynamic findings/characteristic of women with detrusor underactivity.

## Materials and methods

This retrospective cross-sectional study was conducted after the approval of the Institutional Review Board of Buddhist Tzu Chi General Hospital (IRB: 100–06). All the data was anonymized before analysis. The IRB waived the requirement for inform consent.

In our practice, women who visited our urologic clinic due to voiding dysfunction, defined as abnormally slow or incomplete micturition based on symptoms [1], will be educated to have behavioral modification, or given anti-cholinergic/ alpha-blocker according to the patient’s complaints. Those who did not response to first line treatment after 4 weeks period will undergo VUDS to identify the underlying cause of their LUTS. The inclusion criteria of the study were patient who visited our urology out patient clinic during 1997 and 2015, and underwent VUDS after not responding to first line treatment. Patients with high-grade pelvic organ prolapse (Pelvic organ prolapse quantification system stage III and IV) causing anatomical obstruction, acute urinary retention, acute urinary tract infection, and overt central or peripheral neuropathy including spinal cord injury, stroke, Parkinson’s disease, and history of radical pelvic surgery were excluded from the study.

The VUDS was performed by a single experienced urologist and a urodynamic technician who conducted all the urodynamic studies as previously reported[7]. VUDS parameters including first sensation of bladder filling (FSF), full sensation (FS), bladder compliance, maximum flow rate (Q_max_), voiding detrusor pressure at Q_max_ (P_det_), voided volume (VV), post-void residual volume (PVR), cystometric bladder capacity, voiding efficiency (VE, defined as voided volume/bladder capacity x 100), bladder contractility index (BCI, defined as P_det_ + 5 x Q_max_) were measured and recorded.

The following diagnoses were made according to the VUDS findings[8]: DU, detrusor hyperactivity with impaired contractility (DHIC), hypersensitive bladder (HSB), detrusor overactivity (DO), bladder neck dysfunction, cystocele, dysfunctional voiding, poor relaxing external sphincter (PRES), and urethral stenosis. If patients did not have a voiding detrusor contractility of more than 10 cm H2O and needed to void by abdominal straining or were unable to void, detrusor underactivity was diagnosed. HSB was diagnosed when the cystometric bladder capacity was less than 350 mL without evidence of uninhibited detrusor contractions during filling of at capacity. BOO was defined as radiographic evidence of obstruction between the bladder neck and distal urethra in the presence of a sustained detrusor contraction of any magnitude, plus a P_det_ of greater than 35 cmH_2_O in combination with a Q_max_ of less than 15 mL/s[4]. Results were compared with those with normal VUDS parameters. Table 1. listed the diagnostic criteria for each disease.

**Table 1:** 

Diagnosis	Diagnostic criteria
Detrusor underactivity (DU)	patients did not have a voiding detrusor contractility of more than 10 cm H2O and needed to void by abdominal straining or were unable to void
Detrusor overactivity (DO)	urodynamic evidence of spontaneous detrusor contractions occurring during bladder filling (phasic DO) or occurring before uninhibited detrusor contraction voiding at bladder capacity (terminal DO).
Detrusor hyperactivity with impaired contractility DHIC)	DO was associated with incomplete bladder emptying and PVR of more than 100 ml
Hypersensitive bladder HSB)	a strong desire to void at a cystometric bladder capacity (CBC) of less than 350 ml and without occurrence of DO
Bladder outlet obstruction BOO), excluded from our analysis	Include bladder neck dysfunction, cystocele, dysfunctional voiding, poor relaxation of the external sphincter and urethral stenosis

After VUDS, patients with the diagnosis of bladder neck dysfunction, cystocele, dysfunctional voiding, PRES and urethral stenosis were classified as having bladder outlet obstruction and excluded from the final analysis of DU. Patient age, VUDS parameters, and comorbidities were compared among the DU, DHIC, HSB, and DO groups.

### Statistical analysis

The categorical variables are presented as number (proportion), and continuous variables are depicted by mean ± standard deviation. Due to the non-parametric distribution of the urodynamic variables, between group variances were compared using the Welch/Brown analysis, and Games-Howell post hoc test was done to further analyzed the differences between every group. Comorbidities between groups were compared using the Chi-square test. We used scattered-plot diagrams to characterize the relationships between bladder sensation, detrusor contractility, and Q_max_. Spearman correlation was used to determine the correlation coefficients.

## Results

After application of the selection criteria, a total of 1914 women were included in this study. The final diagnoses were normal in 2.9% (n = 56), DU in 23.1% (n = 443), DHIC in 12.0% (n = 231), HSB in 17.0% (n = 325), DO in 2.6% (n = 49) and bladder outlet dysfunction in 42.3% (n = 810). Patient demographics, including age and comorbidities, are summarized in Table 2. In the voiding dysfunction patients, those with normal tracing VUDS and HSB seemed to be younger then those with DU, DHIC and DO. Comorbidities including diabetes mellitus, hypertension, coronary arterial disease, chronic obstructive pulmonary disease, and chronic kidney disease were comparable between each group.

**Table 2:** Patient demographics and comorbidities.

	Normal tracing	bladder dysfunction, 1048(54.7%)				bladder outlet dysfunction	
		DU	DHIC	HSB	DO		P value
Patient No.(%)	56(2.9%)	443(23.1%)	231(12.0%)	325(17%)	49(2.6%)	810(42.3%)	-
Age (years)	53.9(year	66.5(year	75.5(year	53.8(year	67.4(year	59.3(year	<0.01
DM	8(14.3%)	81(18.3%)	49(21.2%)	33(10.2%)	3(6.1%)		<0.01
Hypertension	6(10.7%)	80(18.1%)	49(21.2%)	36(11.1%)	4(8.2%)		0.001
CAD	2(3.6%)	21(4.7%)	10(4.3%)	8(2.5%)	1(2.0%)		0.51
COPD	0(0.0%)	6(1.4%)	2(0.9%)	1(0.3%)	0(0.0%)		0.47
CKD	2(3.6%)	11(2.5%)	6(2.6%)	4(1.2%)	0(0.0%)		0.48

DM: diabetes mellitus, CAD: coronary arterial disease, COPD: chronic obstructive pulmonary disease, CKD: chronic kidney disease, DHIC: detrusor hyperactivity with insufficient contractility, HSB: hypersensitive bladder, DO: detrusor overactivity, DU: detrusor underactivity

Table 3. summarizes the VUDS parameters of each group, and boxplot distribution of the VUDS parameters according to each disease were shown in S1–S3 Figs. The DU group showed an increased threshold for the FSF and FS compared with those in the DHIC, HSB, and DO groups. The cystometric bladder capacity of the DU group was significantly larger than in the DHIC, HSB, and DO groups but was significantly smaller than in the normal VUDS group. In addition, women in the DU group had the lowest P_det_, BCI, Q_max_, and the largest PVR. Decreased VV and VE were also noted in the DU group when compared to the normal, HSB, and DO groups. There was no significant difference in the bladder compliance among groups.

**Table 3:** Urodynamic parameters of all subgroups.

	Normal tracing (n = 56)	DU (n = 443)	DHIC (n = 231)	HSB (n = 325)	DO (n = 49)	Bladder outlet dysfunction (n = 810)	P value
FSF(ml)	166.8±71.6	198.3±108.6	139.1±72.3	117.8±53.7	130.9±70.0	141.3±69.1	<0.01
FS(ml)	290.3±103.0	291.5±129.6	195.6±100.7	193.5±72.0	185.7±91.6	222.9±97.4	<0.01
Compliance	84.8±72.1	65.6±91.6	58.3±68.8	66.2±69.3	51.0±54.8	71.7±84.8	0.07
Pdet(cmH2O)	17.3±8.23	7.6±9.59	17.9±9.42	18.7±9.56	20.2±10.1	33.3±23.1	<0.01
Qmax(ml/s)	24.1±7.81	4.6±5.36	6.3±4.41	12.3±5.85	12.9±8.33	8.8±6.21	<0.01
CQmax	1.09±0.38	0.25±0.30	0.37±0.24	0.76±0.34	0.82±0.45	0.51±0.35	<0.01
Vol.(ml)	488.6±114.3	107.6±120.7	103.9±82.1	234.6±86.4	230.1±124.4	202.4±141.7	<0.01
PVR(ml)	19.4±28.8	285.4±199.7	195.3±143.0	34.3±75.3	28.0±40.6	126.3±139.2	<0.01
Capacity(ml)	508.1±119.9	393.1±167.4	299.2±156.3	268.9±89.8	258.1±126.0	328.7±149.9	<0.01
VE(%)	96.4±4.94	31.0±33.2	36.4±22.4	87.5±19.6	87.6±13.7	62.6±32.3	<0.01
BCI	138.0±40.1	30.6±30.2	49.6±22.9	80.7±32.3	85.1±40.9	77.7±38.6	<0.01

FSF: first sensation of filling, FS: full sensation, Pdet: voiding detrusor pressure at Qmax, Qmax: maximum flow rate, CQmax: corrected Qmax, Vol.: voided volume, PVR: post-void residual volume, VE: voiding efficiency, BCI: bladder contractility index, DHIC: detrusor hyperactivity with insufficient contractility, HSB: hypersensitive bladder, DO: detrusor overactivity, DU: detrusor underactivity

Data analyzed with Welch/Brown analysis, and Games-Howell post-hoc test. P value< 0.05 as significant.

The urodynamic parameters, including bladder sensation, P_det_, and PVR, were similar between HSB and DO groups. Increased bladder sensation (decreased threshold of FSF and FS), while decreased bladder capacity, lower Q_max_, and BCI were noted compared to the normal group. There were no significant differences in the bladder capacity, PVR, or VE between the normal group and HSB or DO groups. As previously noted, the Q_max_ was positively correlated with the voiding volume, and corrected Q_max_ (CQ_max_, defined as Q_max_/VV^1/2^) was used to evaluate the Q_max_ over a wide range of voiding volumes[9]. CQ_max_ was calculated and compared between the HSB, DO, and normal groups to see if a smaller voided volume resulted in a lower Q_max_ in the HSB and DO groups. However, the CQ_max_ was still significantly lower in the HSB and DO groups compared to the normal group.

**Fig 1:**
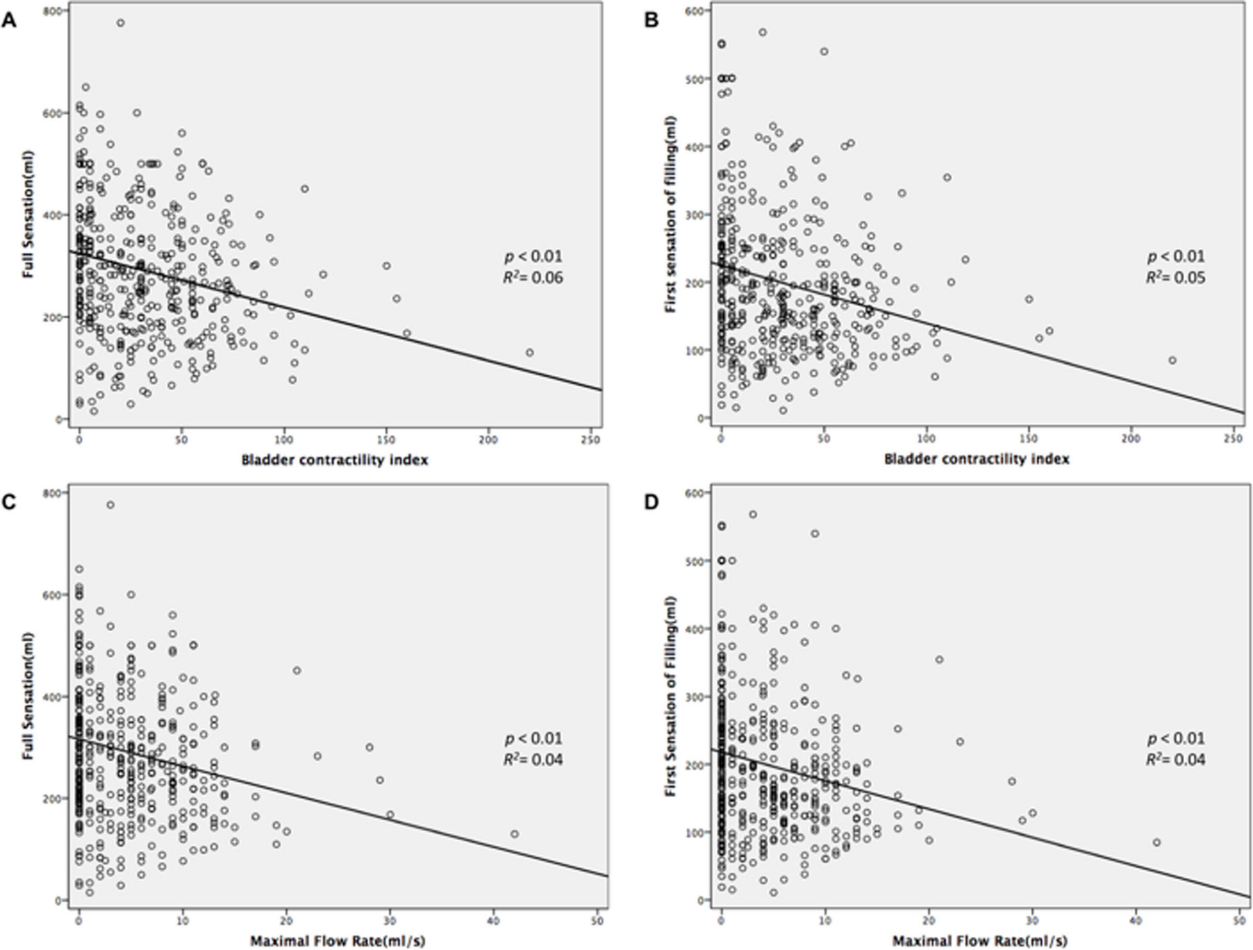
The negative association between bladder contractility and bladder sensation in women with detrusor underactivity. (A) Bladder contractility index (BCI) vs. full sensation (FS). (B) BCI vs. the first sensation of filling (FSF). (C) Maximum flow rate (Q_max_) vs. FS. (D) Q_max_ vs. FSF.

Linear regression analysis showed that bladder sensation (including FSF and FS) of the DU patients was negatively associated with bladder contractility (FSF vs. BCI, r^2^ = - 0.05; FS vs. BCI, r^2^ = 0.06; both p-value < 0.01) and Q_max_ (FSF vs. Q_max,_ r^2^ = 0.04; FS vs. Q_max_, r^2^ = 0.04; both p-value < 0.01) (Fig 1A–1D). In addition, we further stratified the patients in the DU group into very low detrusor pressure (P_det_ < 5 cmH_2_O) and low detrusor pressure (P_det_ ≥ 5 cmH_2_O), and compared these two subgroups in terms of age, urodynamic parameters, and comorbidities (Table 4). There were 162 patients in the very low detrusor pressure group, and 281 patients in the low detrusor pressure group. The mean age was similar in both groups. Bladder sensation, including FSF and FS, were significantly higher in the very low detrusor pressure group. Patients in the very low detrusor pressure group also had lower Q_max_, VV, VE, BCI, and higher PVR. The prevalence of diabetes mellitus was significantly higher in the very low detrusor pressure group.

**Table 4:** Subgroup analysis of the patients with detrusor underactivity.

	Very low detrusor pressure (Pdet < 5 cmH_2_O)	Low detrusor pressure (Pdet ≥ 5 cmH_2_O)	P value
Patient No (%)	162 (37%)	281 (63%)	
Age (years)	67.9 ± 14.2	66.0 ± 14.5	0.61
FSF(ml)	230 ± 127	180 ± 91.4	<0.01
FS(ml)	326 ± 144	273 ± 116	<0.01
Compliance	76.1±104	59.5 ± 82.8	0.11
Pdet (cmH2O)	0.8 ± 1.22	10.6 ± 7.16	<0.01
Qmax (ml/s)	2.4 ± 4.06	5.8 ± 5.66	<0.01
VV (ml)	55.1 ± 91.0	138.6 ± 126.0	<0.01
PVR (ml)	364.7 ± 224.3	241.2 ± 168.9	<0.01
Capacity (ml)	419.9 ± 198.7	379.9 ± 144.3	<0.01
VE (%)	17.7 ± 29.2	33.4 ± 33.8	<0.01
BCI	13.1 ± 20.7	40.0 ± 29.8	<0.01
DM	42 (25.9%)	38 (13.8%)	<0.01
Hypertension	33 (20.4%)	46 (16.7%)	0.33
CAD	10 (6.2%)	11 (4.0%)	0.3
COPD	1 (0.6%)	5 (1.8%)	0.29
CKD	7 (4.3%)	4 (1.4%)	0.06

FSF: first sensation of filling, FS: full sensation, Pdet: voiding detrusor pressure at Qmax, Qmax: maximum flow rate, VV.: voided volume, PVR: post-void residual volume, VE: voiding efficiency, BCI: bladder contractility index detrusor overactivity, DM: diabetes mellitus, CAD: coronary arterial disease, COPD: chronic obstructive pulmonary disease, CKD: chronic kidney disease,

## Discussion

The results of this study revealed that DU, DHIC, HSB, and DO contribute to voiding dysfunction in women. Women with DU had reduced bladder sensation compared to patients with DHIC, HSB, and DO. Reduced bladder sensation is associated with greater FSF, FS, and larger cystometric bladder capacity in DU patients. In the DU patients, the bladder sensation was negatively associated with detrusor contractility (P_det_ and BCI) and Q_max_.

The cohort of this study includes both populations of “intrinsic” bladder dysfunction and bladder outlet obstruction. The value of urodynamic study in differentiating bladder outlet dysfunction had been well characterized in our previous study[10]. But this analysis focused on the subgroup of the intrinsic bladder dysfunction, especially for those with detrusor underactivity, which was completely different from the previous work.

Previously, detrusor or neurogenic etiologies were considered to contribute to DU [11–13]. However, normal sensory signaling and afferent transduction are essential for initiating micturition and maintaining detrusor contractility[14]. Recently, urotheliogenic etiologies have emerged as important factors for DU. Phillip et al. conducted a retrospective study and found that in non-neurogenic, non-obstructed DU patients, decreased central sensitivity to volume afferent activity may play a role[15]. Animal studies have also shown preserved detrusor expulsive strength with impaired sensations of bladder filling in aging mouse[14]. At the molecular level, down-regulated sensory protein expression was also found in the DU patients[16]. We found that patients in the DU group had increased thresholds of FSF and FS compared to the normal group and other voiding dysfunctional groups. These thresholds suggest reduced bladder sensation may be specific to the etiology of DU. The negative association between detrusor contractility and bladder sensation in the linear regression and increased thresholds of FSF, FS, and bladder capacity in the very low detrusor pressure DU group further support this hypothesis. Although patients in the DHIC group also had insufficient detrusor contractility and even urinary retention, their bladder sensation did not decrease. In contrast, the threshold of FSF and FS in the DHIC, HSB, and DO groups significantly decreased to a different level compared to the normal group. Patients with DHIC might have increased afferent sensitivity and impaired detrusor contractility resulting from different neurogenic or myogenic mechanisms, respectively.

DU is a multi-factorial symptom complex. Other than the bladder itself, aging and comorbidities also contribute to this condition. As previously mentioned, aging may directly cause diminished detrusor contractility or impaired afferent signaling[14]. In the present study, the age in the DU group was significantly older than that of the normal and HSB groups but younger than that of the DHIC group. Diabetic bladder dysfunction involves both the storage and voiding phases. In the early phase, hyperglycemia-induced polyuria results in bladder hypertrophy, causing neurogenic and myogenic alteration. In the later phase, the accumulative oxidative stress leads to decompensated bladder, i.e., DU[17]. In the current study, the prevalence of diabetes mellitus did not differ between the DU and normal groups. The prevalence of diabetes mellitus in general population of Taiwan is rather high (7.1% in 2013)[18]. It is possible that the diabetes mellitus patients in the normal VUDS group were in the early phase of diabetes mellitus; therefore, although they had LUTS, the VUDS results were still normal. Other comorbidities have been postulated to contribute to DU by affecting bladder perfusion, causing chronic bladder ischemia or via the renin-angiotensin system[19–21]. However, in the present study, there was no significant difference between the DU group and the normal group for these comorbidities.

One of the reasons that DU is overlooked is the difficulty of diagnosing it without invasive urodynamic studies. Underactive bladder may be considered a clinical diagnosis, parallel to that of overactive bladder, with DU being one of the urodynamic findings. According to the ICS definition of DU, in addition to the requirement of urodynamic studies, there are no standardized criteria for the normal duration of detrusor contraction, and a normal time span of micturation, which further hampered its clinical diagnosis. One recent study tried to correlate DU by clinical symptoms and signs without invasive pressure-flow studies[22]. The study applied strict criteria to the diagnose DU, BOO, and normal pressure flow to prevent overlapping. The investigators found that clinical symptoms of LUTS correlated poorly with the final diagnosis[4]. In addition to BOO and normal pressure-flow studies, various subtypes of bladder dysfunction need to be differentiated from others.

We believe that an accurate diagnosis of voiding dysfunction through VUDS is crucial. In this study, all patients with voiding dysfunction in whom the initial medical treatment failed then underwent VUDS. Among them, BOO was diagnosed in 48.7%, including bladder neck dysfunction, dysfunctional voiding, PRES, and urethral stenosis. Without VUDS, it is not possible to identify the site of BOO. In the present study, DU patients were characterized by decreased bladder sensation, very low P_det_, Q_max_, VE, and BCI on VUDS. Their bladder capacity was smaller than the in the normal group but larger than in other voiding dysfunction groups. In contrast, the DHIC, HSB, and DO groups had increased bladder sensation and normal P_det_. The DHIC group also exhibited high PVR, low VE, and BCI, while those parameters remained normal in the HSB and DO groups.

Although patients with HSB and DO usually present with storage LUTS rather than voiding LUTS. In this study, we found that 19.6% of women with voiding dysfunction had HSB or DO. These patients usually voided a small urine volume and felt difficulty initiating or terminating urination, resulting in voiding LUTS. Patients with voiding dysfunction and a diagnosis of HSB or DO have lower Q_max_. Initially, we thought this might be due to the smaller voided volume; however, they also have lower CQ_max_ after adjustment of the volume factor. Compared to the normal group, the HSB and DO groups had lower BCI, which could partly explain this phenomenon. A previous study has shown that the obstruction percentage (defined as Q_max_ < 15ml/s and P_det_ Q_max_ > 40 cmH_2_O) was significantly higher in those with idiopathic DO than in the control group (36% versus 15%, p<0.001)[23]. Additionally, increased detrusor pressure upon urethral opening and closure were observed in patients with detrusor instability[24]. Both factors may contribute to the lower CQ_max_ in the HSB and DO groups. Further study is needed to clarify the etiology of these disorders.

Finally, the urodynamic parameters in the HSB and DO groups did not differ significantly. Afferent noise resulting in hypersensitive bladder is one of the major factors causing overactive bladder[25]. The urodynamic parameters of HSB and DO include increased bladder sensation and normal P_det_ compared to the normal group, indicating the urothelium could also contribute to its pathophysiology. Alterations in sensory receptors and enzymes of the bladder mucosa in rats with metabolic syndrome elicit bladder oversensitivity and overactive bladder[26]. The results of our study further support that the two disease entities could share a common pathophysiology.

The strengths of this study include the large sample size of the patients with various bladder dysfunctions, and the VUDS performed similarly throughout the study period. The limitations of the study include the retrospective nature and insufficient information on the severity and duration of diabetes mellitus in the patients. In addition, during the long study period, there might be some changes in clinical practice due to the innovation of medication. However, the basic principle of first line management of the voiding dysfunction patient remained the same, and all the diagnostic definition and VUDS techniques followed the guideline of the ICS, which might minimize the impact on the results.

## Conclusion

The results of this study clarify the urodynamic characteristics of various bladder dysfunctions in women with voiding disorders. Reduced bladder sensation was found specifically in the DU patients along with a negative association between the detrusor contractility and bladder sensation. Low Q_max_ and BCI were also noted in the HSB and DO patients. VUDS plays a crucial role in classifying different types of bladder dysfunction, which assist in making a subsequent treatment plan for women with voiding dysfunction.

## Supporting information

S1 Fig Distribution of FSF, FS, Compliance and Pdet according to different diseases. (TIFF)

S2 Fig Distribution of Qmax, cQmax, Vol and PVR according to different diseases. (TIFF)

S3 Fig Distribution of capacity, VE and BCI according to different diseases. (TIFF)
