# Supplementary figures and images for: Urodynamic characteristics of detrusor underactivity in women with voiding dysfunction
Source: PLoS One. 2018 Jun 20;13(6):e0198764. doi: 10.1371/journal.pone.0198764 (PMC6010249; doi:10.1371/journal.pone.0198764)

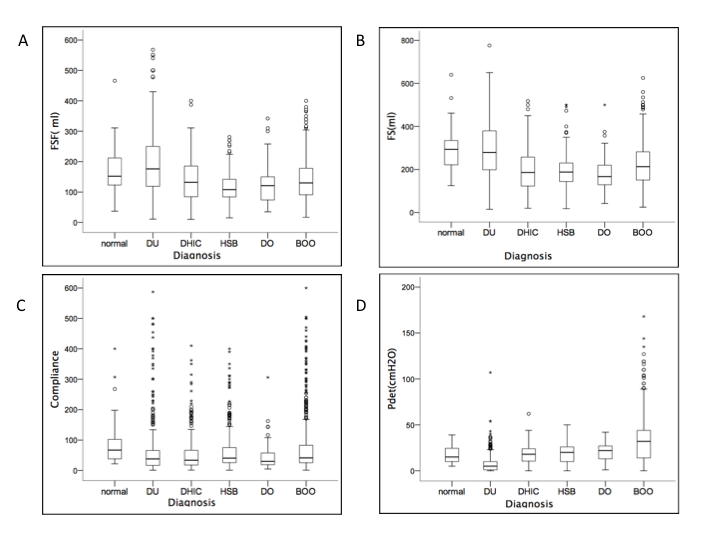

Supplement: S1 Fig — (TIFF) [file pone.0198764.s001.tiff]

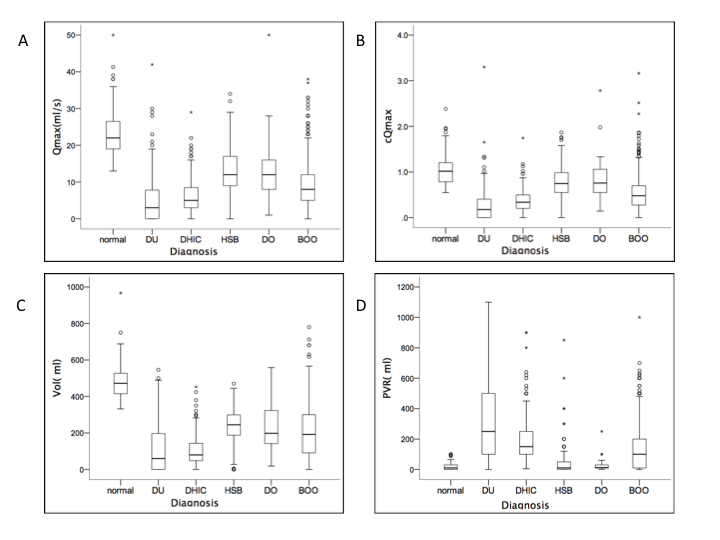

Supplement: S2 Fig — (TIFF) [file pone.0198764.s002.tiff]

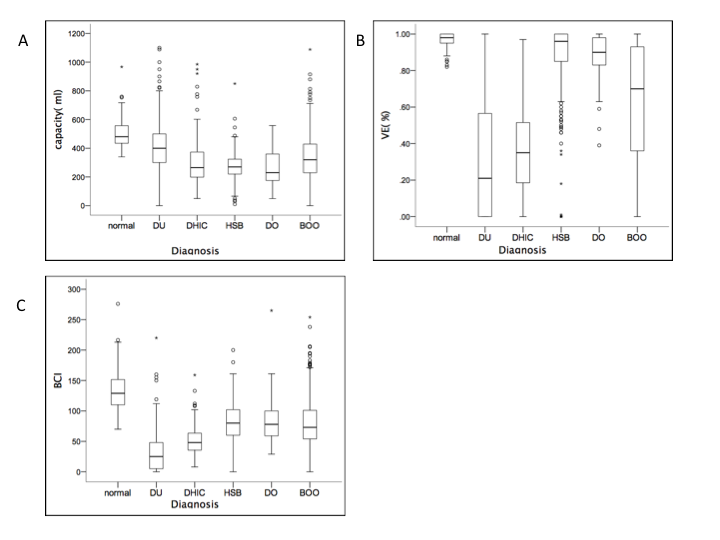

Supplement: S3 Fig — (TIFF) [file pone.0198764.s003.tiff]
